# Role of redox environment on the oligomerization of higher molecular weight adiponectin

**DOI:** 10.1186/1471-2091-12-24

**Published:** 2011-05-23

**Authors:** David B Briggs, Rebecca M Giron, Pamela R Malinowski, Martha Nuñez, Tsu-Shuen Tsao

**Affiliations:** 1 Department of Chemistry and Biochemistry, University of Arizona, 1656 E Mabel St MRB 430J, Tucson, AZ, 85724, USA

## Abstract

**Background:**

Adiponectin is an adipocyte-secreted hormone with insulin-sensitizing and anti-inflammatory actions. The assembly of trimeric, hexameric, and higher molecular weight (HMW) species of adiponectin is a topic of significant interest because physiological actions of adiponectin are oligomer-specific. In addition, adiponectin assembly is an example of oxidative oligomerization of multi-subunit protein complexes in endoplasmic reticulum (ER).

**Results:**

We previously reported that trimers assemble into HMW adiponectin *via *intermediates stabilized by disulfide bonds, and complete oxidation of available cysteines locks adiponectin in hexameric conformation. In this study, we examined the effects of redox environment on the rate of oligomer formation and the distribution of oligomers. Reassembly of adiponectin under oxidizing conditions accelerated disulfide bonding but favored formation of hexamers over the HMW species. Increased ratios of HMW to hexameric adiponectin could be achieved rapidly under oxidizing conditions by promoting disulfide rearrangement.

**Conclusions:**

Based upon these observations, we propose oxidative assembly of multi-subunit adiponectin complexes in a defined and stable redox environment is favored under oxidizing conditions coupled with high rates of disulfide rearrangement.

## Background

The adipocyte-secreted hormone adiponectin contributes to the maintenance of whole body insulin action and normal cardiovascular and endothelial functions [1-7]. Adiponectin homo-oligomerizes into different isoforms: trimer, hexamer and several higher molecular weight (HMW) species, the largest and the most abundant of which is the octadecamer [8-10]. Trimeric adiponectin, the basic building block of all oligomers, is held together by strong hydrophobic interactions among the three monomers in the globular head domain and by triple helical coils in the collagenous domain [11]. The HMW adiponectin species are decreased in insulin resistance to a larger extent than the other isoforms [12-16]. In contrast, treatment with thiazolidinediones (TZD), a class of drugs that are used for treatment of type 2 diabetes, increases HMW adiponectin concentration in circulation [16,17]. Changes in gene expression alone could not account for TZD-stimulated increase in HMW adiponectin levels [17], therefore it is important to understand the mechanisms that impair the formation of HMW adiponectin in adipocytes.

Disulfide bonds play an important role in the oligomerization of adiponectin. Adiponectin contains two conserved cysteines: one in the globular head of adiponectin and the one at the N-terminal portion [10]. Previous studies have shown that the non-solvent accessible cysteine in the globular head of adiponectin does not influence adiponectin oligomerization [10], whereas the cysteine near the N-terminus (C22 residue in mature murine adiponectin) mediates oligomerization into the hexamer and octadecameric isoforms of adiponectin [10,18,19]. The trimeric isoform of adiponectin contains a dimer linked by an intra-trimer disulfide bond and a monomer [10]. In contrast, the cysteines in N-terminal regions of adiponectin hexamers and octadecamers are fully oxidized as disulfides [10]. In previous studies, mutation of C22 to either alanine or serine precludes formation of the higher-ordered oligomers [10,18,19]. Intriguingly, recent gel filtration chromatography showed that large complexes of adiponectin could exist following treatment with reducing agents [20]. Using high resolution gel electrophoresis, dynamic light scattering, and collision-induced dissociation nano-electrospray ionization mass spectrometry, we showed definitively that octadecameric HMW adiponectin is stable in absence of disulfide bonds [21]. We addressed this paradox in our previous report by demonstrating that although disulfide bonds are not required for stability of the mature HMW species, they are necessary for oligomerization [21]. Disulfide bonds likely provide the covalent linkages needed to stabilize intermediate oligomers in the step-wise addition of subunits during the expansion of oligomers. Consistent with this conclusion, fully oxidized adiponectin hexamers lacking free thiols assembled into octadecamers at an extremely slow rate when compared with that of reduced trimers undergoing disulfide formation during assembly [21].

Given the importance of disulfide bonds in the assembly of HMW adiponectin, we hypothesized that redox conditions can affect the distribution of adiponectin oligomers through changes in the rate of and pattern of disulfide formation. To test this hypothesis, we monitored oligomerization of adiponectin under various redox conditions. Under conditions with low reduction potential, adiponectin did not undergo oxidative assembly in a physiologically relevant time frame. Under oxidizing conditions, the predominant oligomers were hexamers and trimers that oligomerized poorly into larger species. We found that conditions favoring rearrangement of disulfide bonds led to robust and relatively rapid formation of HMW adiponectin. Taken together, our data provide a redox-based model for oxidative assembly of a multi-subunit adiponectin protein complex and may implicate ER redox environment alteration as a contributing factor in decreased levels of HMW adiponectin in insulin-resistant disease states. Our data also provide potential mechanisms for understanding the previously observed effects of the ER oxidoreductase Ero1-Lα and chaperones DsbA-L and ERp44 on adiponectin production [22-24].

## Results

### Effect of slower oxidation rate on oligomer distribution during adiponectin re-oligomerization

**Figure 1:**
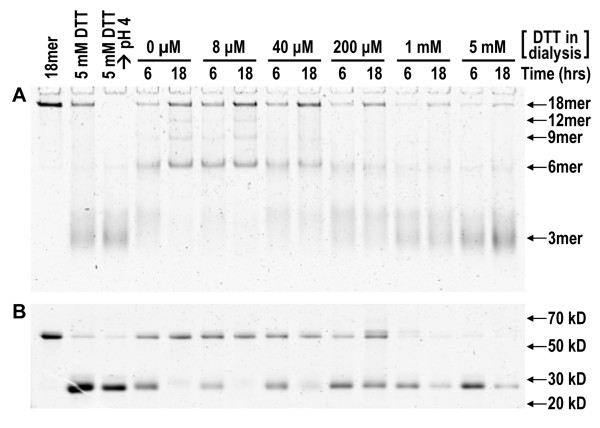
**Re-oligomerization of adiponectin following dialysis into defined concentrations of DTT**. **(A) **Native PAGE of adiponectin oligomers after collapse to trimers and subsequent re-oligomerization as concentrations of DTT were equilibrated from 5 mM to 0.008, 0.04, 0.2, 1, or 5 mM by dialysis over an 18-hr period. **(B) **Non-reducing denaturing SDS-PAGE analysis to identify the oxidation state of adiponectin as either in the disulfide-bonded dimer state or reduced monomer state in each of the different conditions. Oligomerization and oxidation states were examined at 6 and 18 hrs. The redox state of adiponectin was fixed by adding NEM to the sample at the end of reassembly reactions. 3 mer: trimer; 6 mer: hexamer; 9 mer: nonamer; 12 mer: dodecamer; 18 mer: octadecamer. The number of monomers in trimers, hexamers, and octadecamers in native gels were independently verified using native mass spectrometry and gel filtration chromatography (data not shown). The number of monomers in nonamers and dodecamers were determined by extrapolation of migration distances of known oligomers in native gels as described in Methods.

We previously showed the main function of disulfide bonds in oxidative oligomerization of adiponectin is to stabilize intermediate oligomers and that fully oxidized hexamers oligomerized to HMW adiponectin at a much slower rate than reduced trimers [21]. This led us to hypothesize that adiponectin oligomerization into HMW or hexameric isoforms could be modulated by the prevailing redox environment. Specifically, our model predicts that oxidizing conditions will accelerate disulfide bonding and favor the formation of fully oxidized hexamers that are unable to assemble further into HMW adiponectin. To test this hypothesis, we performed re-oligomerization experiments in which the rate of disulfide formation was slowed by decreasing the rate of DTT removal. Following collapse to trimers by reduction with 5 mM DTT and lowering of pH to 4, reassembly of HMW adiponectin was examined as reactions were exchanged into varying concentrations of DTT in PBS at pH 7.4. As reactions were dialyzed into increasing concentrations of DTT, decreased formation of both octadecameric and hexameric adiponectin were observed, with hexamer formation affected to a greater extent than octadecamer formation (Figure 1A). The increased susceptibility of hexamers to reduction could be explained by the inherent stability of reduced octadecamer [21]. Alternatively, the inter-trimer disulfide bonds between hexamers are more susceptible to reduction than those in the HMW complex [18]. Formation of octadecamers and hexamers was accompanied by oxidation of reduced monomers to disulfide-bonded dimers (Figure 1B). As the concentration of DTT increased, the amount of reduced monomer increased at the expense of oxidized dimer, indicating reduced rates of disulfide formation (Figure 1B). This increase in reduced monomers compared to oxidized dimers is accompanied by a decrease in the conversion of trimers to larger species (Figure 1A). These results demonstrate that decreased rate of disulfide bonding led to decreased formation of both HMW and hexameric adiponectin formation. Complete removal of DTT did not inhibit HMW adiponectin formation at the expense of hexamers (Figure 1A). These results do not support the notion that modulating the rate of disulfide bond formation could result in preferential formation of HMW adiponectin over hexamers or *vice versa*.

### Defining the reduction potential of adiponectin oligomerization

**Figure 2:**
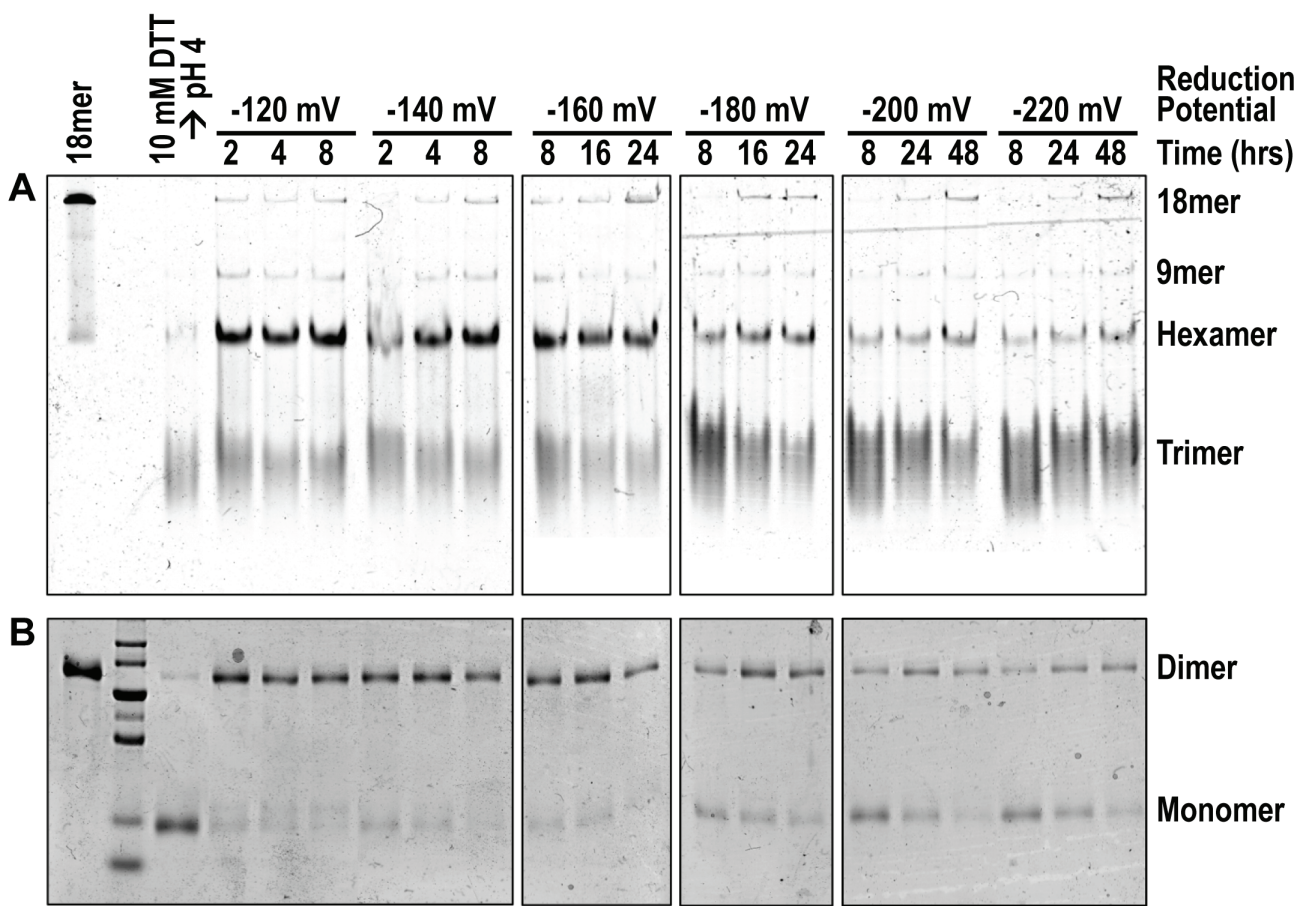
**Redox titration of adiponectin oligomerization in glutathione-based redox buffers**. **(A) **Native PAGE of adiponectin oligomers after collapse to trimers and subsequent incubation in various reduction potentials and time points as indicated. **(B) **Non-reducing denaturing SDS-PAGE analysis of disulfide-bonded dimers or reduced monomers at various time points. Purified bovine octadecameric adiponectin was collapsed to trimers as described in Methods. After collapse, different ratios of oxidized and reduced glutathione at reduction potentials ranging from -120 to -220 mV were added and allowed to incubate in an anaerobic chamber for up to 48 hrs. The total concentration of glutathione in each condition ranged from 1 to 3 mM. At each time point, aliquots were removed and further disulfide bond exchange was halted by treatment with NEM.

To better define the role of redox environment on adiponectin oligomerization, we performed re-oligomerization experiments in a series of glutathione-based redox buffers with varying reduction potentials bracketing the reported ER reduction potential of -180 mV [25]. In these experiments, DTT added to reduce purified octadecamers was removed prior to start of reassembly reactions by addition of oxidized glutathione. For each reduction potential, pilot experiments were first performed to ensure sufficient time was given to allow adiponectin redox state to reach equilibrium (data not shown). To minimize the effect of oxygen on oxidation, DTT removal and subsequent re-oligomerization assays were carried out in an anaerobic chamber. In contrast to the results in Figure 1 in which re-assembly was allowed to proceed by slow and gradual removal of reducing agents, in these experiments reducing agents were removed before re-oligomerization began by incubation in glutathione-based buffers, resulting in decreased levels of HMW adiponectin relative to hexamers or trimers under all redox conditions tested (Figure 2A). Oxidation of adiponectin reached equilibrium faster and to a larger extent as the reduction potential increased from -220 to -120 mV (Figure 2B). At -220 and -200 mV, the rate of adiponectin oxidation was slowest and the concentrations of reduced monomers the highest among the reduction potentials tested (Figure 2B). Trimers predominated under these conditions (Figure 2A). At -140 and -120 mV, nearly all adiponectin molecules became disulfide-bonded within 2 hrs of incubation and majority of reassembled oligomers were hexamers (Figure 2A and 2B). At -180 and -160 mV, the rate and extent of adiponectin oxidation were moderate compared with either extremes, yet the predominant oligomers were trimers or hexamers with small amounts of HMW adiponectin upon prolonged incubation (Figure 2A and 2B). These data indicate that in the physiologically relevant glutathione redox buffer with reduction potentials ranging from -220 to -120 mV, oxidation was either too slow to promote significant formation of HMW adiponectin in a reasonable time frame or too fast to allow intermediates to grow by forming additional disulfide bonds before becoming completely oxidized.

### Inability of adiponectin trimers to oligomerize under highly oxidizing conditions

One potential explanation for lack of HMW adiponectin formation in glutathione based redox buffers is incomplete oxidation of adiponectin. Even at -120 mV reduction potential, significant amounts of adiponectin monomers remained (Figure 2B). These monomers could be in either the glutathionylated or free thiol forms, and likely form the trimers that were also present at significant levels at -120 mV (Figure 2A). To assure that the reduction potential was high enough to completely oxidize adiponectin, we repeated adiponectin reassembly experiments at reduction potentials as high as +40 mV, and found that hexamers were still the predominant species and small amounts of adiponectin monomers remained (Additional file 1: Figure S1A and S1B).

**Figure 3:**
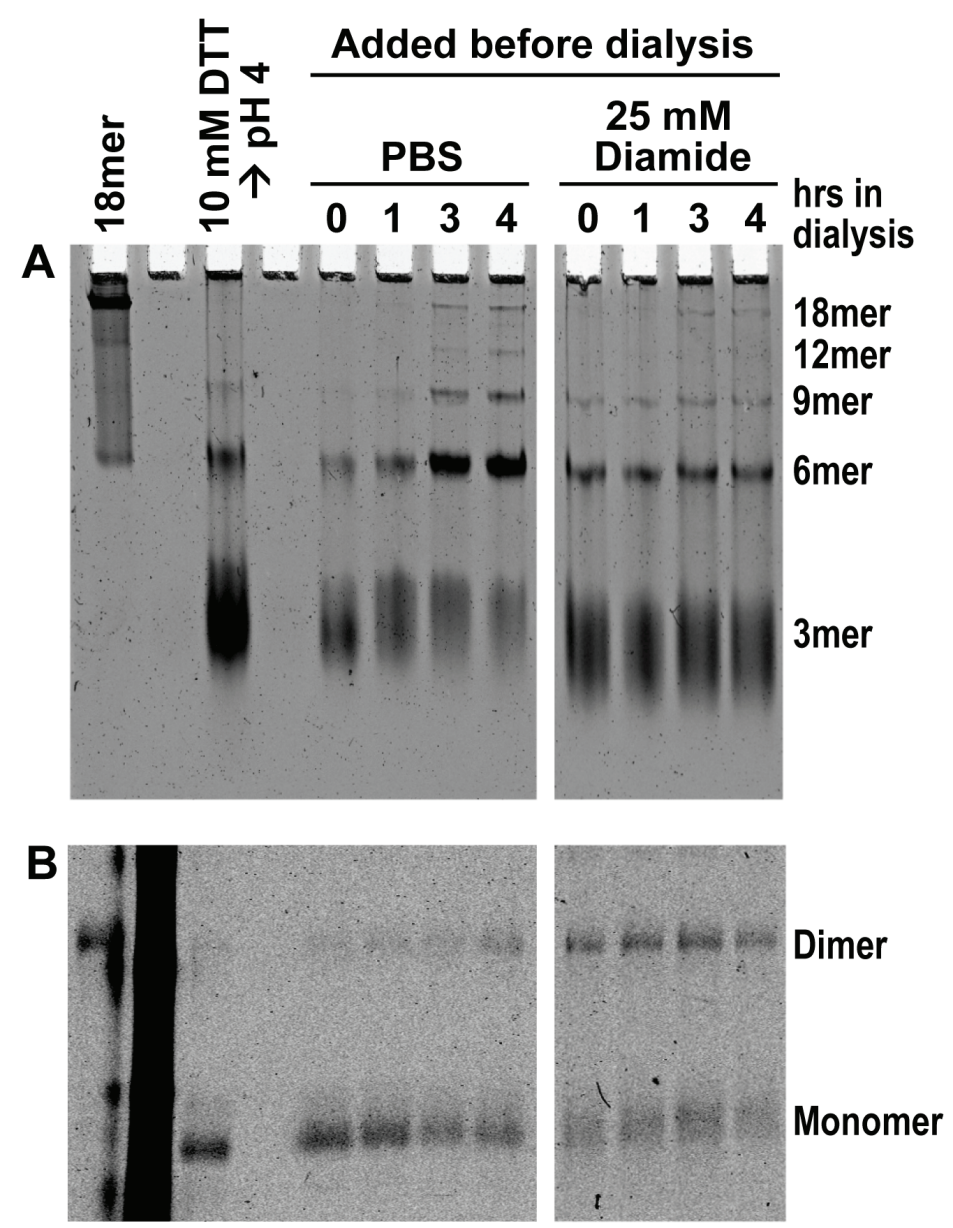
**Re-oligomerization of adiponectin following diamide treatment**. **(A) **Native PAGE of adiponectin oligomers after treatment with diamide followed by dialysis into PBS. **(B) **Non-reducing SDS-PAGE analysis of oxidized dimers and reduced monomers at indicated time points. Bovine octadecameric adiponectin was collapsed to trimers as described in Methods. Subsequently, the samples were treated with PBS or 25 mM diamide and allowed to dialyze against PBS for the indicated amount of time. To fix the redox state of adiponectin, NEM was added and stored at 4°C until analysis.

Adiponectin monomers that remained at high reduction potentials might be glutathionylated. To address the possibility that glutathionylated adiponectin has a higher reduction potential than disulfide-bonded adiponectin, we used diamide, which oxidizes adiponectin without mixed disulfide formation, as the oxidizing agent in our re-oligomerization reactions. As shown in Figure 3, 25 mM diamide added to reduced trimers failed to completely oxidize adiponectin, as evidenced by abundant monomers following diamide treatment (Figure 3B), and resulted in much of the adiponectin trapped in trimers rather than proceeding to HMW adiponectin (Figure 3A). These data suggest that the remaining cysteine in partially oxidized adiponectin trimer (consisting of a disulfide bonded dimer and a reduced monomer) has an unusually high reduction potential; very tiny amounts of reduced glutathione are all that is required to keep it reduced. If the remaining cysteine in a partially oxidized trimer is buried and inaccessible to solvent, it could result in it having a high reduction potential. Partially oxidized adiponectin trimer may exist in a conformation that precludes oligomerization, which could contribute to impaired assembly of HMW adiponectin under highly oxidizing conditions.

### Facilitation of HMW adiponectin by disulfide rearrangement

**Figure 4:**
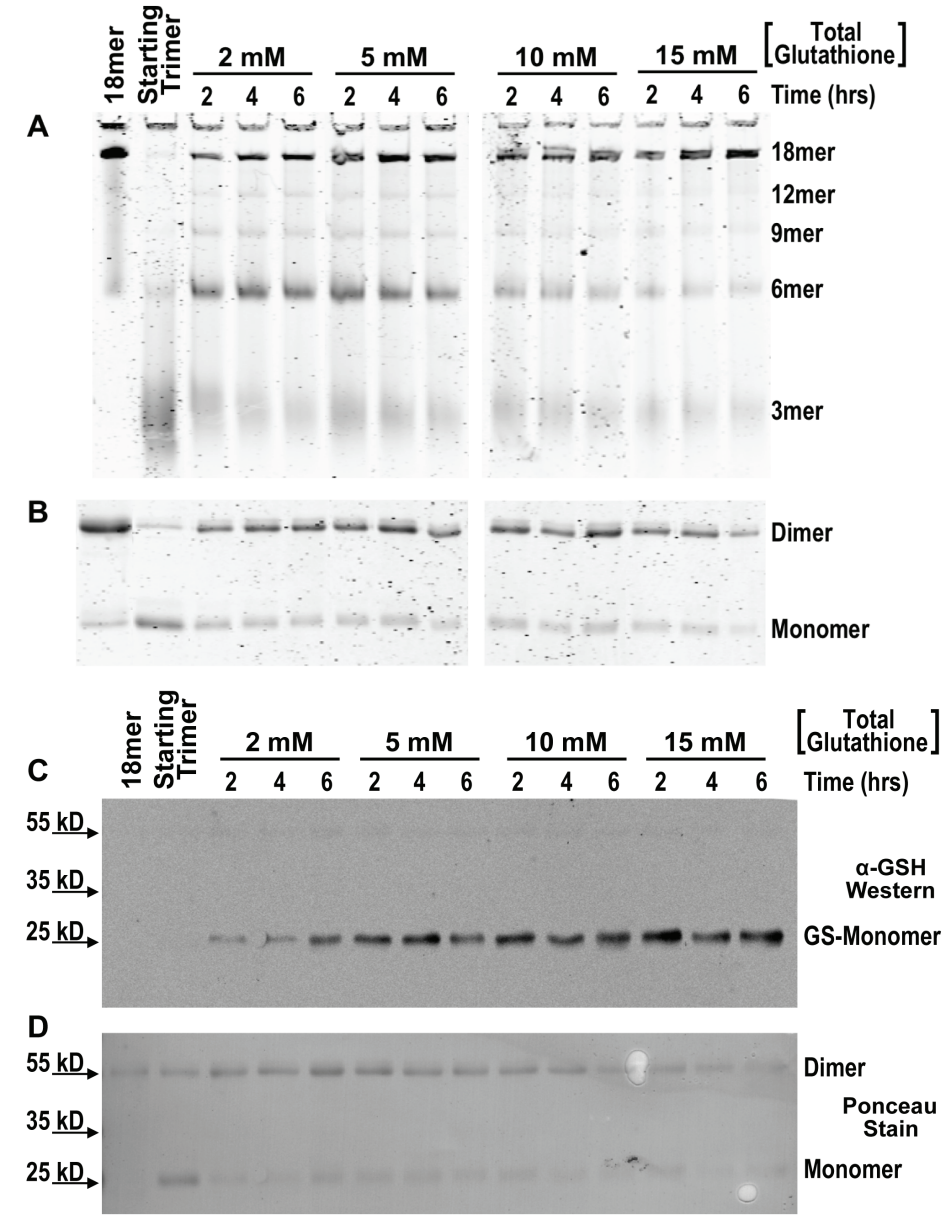
**Re-oligomerization of adiponectin in 2, 5, 10, and 15 mM total glutathione at a constant reduction potential of -120 mV**. **(A) **Native PAGE of adiponectin oligomers after collapse and subsequent incubation in various concentrations of glutathione kept at a reduction potential of -120 mV. **(B) **Non-reducing denaturing SDS-PAGE analysis of disulfide-bonded dimers and reduced monomers of the same reactions. **(C) **Western blot analysis of adiponectin glutathionylation under non-reducing denaturing conditions. Anti-glutathione primary antibody was purchased from Arbor Assays. **(D) **Ponceau S stain of the blot in C prior to blocking. Purified bovine octadecamer was collapsed to trimers as described in Methods. After collapse and removal of DTT, adiponectin was allowed to re-oligomerize in the presence of 2, 5, 10, or 15 mM total glutathione concentrations held at a constant reduction potential of -120 mV. Experiments were performed in anaerobic conditions to minimize the oxidizing effects of atmospheric oxygen. At each time point, NEM was added prior to removal of samples from anaerobic chamber. α-GSH: anti-GSH antibody; GS-Monomer: glutathionylated monomer.

The data presented above indicate that oxidation of reduced adiponectin trimers proceeds faster under oxidizing conditions, and that under these conditions hexamers are the predominant adiponectin oligomer. However, assembly of HMW adiponectin could occur over time in absence of net adiponectin oxidation under many conditions tested. In Figure 2, HMW adiponectin accumulated over time at -160 mV without overt changes in the ratio of dimers to monomers, suggesting that adiponectin oligomerization could proceed under oxidizing conditions *via *disulfide rearrangement. We therefore tested whether increasing concentrations of glutathione at a fixed redox potential could enhance adiponectin oligomerization by promoting disulfide rearrangement. Increasing concentrations of total glutathione but maintaining the reduction potential constant at -120 mV led to increased ratios of HMW versus hexameric adiponectin (Figure 4A). In reactions with higher total glutathione concentrations, the increased levels of octadecamers were associated with decreased levels of hexamers and trimers (Figure 4A). The levels of adiponectin dimers and monomers did not significantly change with increasing concentrations of total glutathione (Figure 4B), indicating net oxidation states were not altered by glutathione levels. Additionally, glutathionylation of adiponectin increased with higher concentrations of total glutathione, consistent with increased disulfide rearrangement (Figure 4C and 4D).

**Figure 5:**
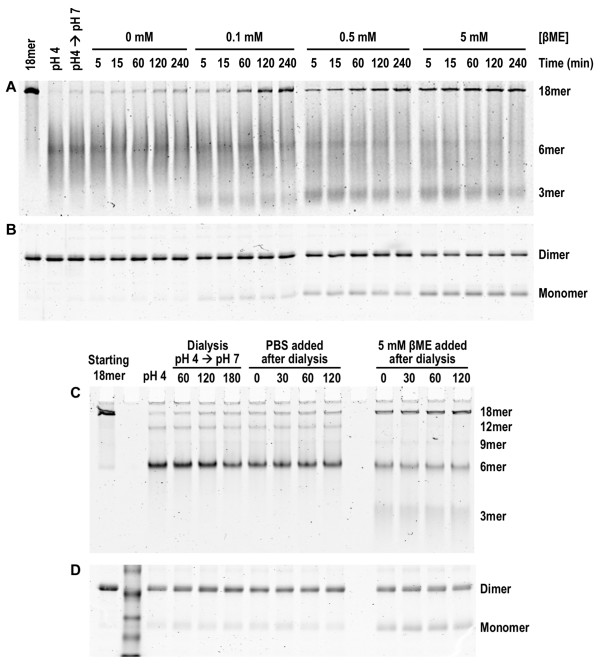
**Re-oligomerization of hexameric adiponectin after treatment with βME**.**(A) **Native PAGE of adiponectin oligomers after collapse of octadecamers to hexamers at pH 4 and subsequent neutralization in Tris-buffer and treatment with various concentrations of βME. **(B) **Non-reducing denaturing SDS-PAGE analysis of adiponectin dimers and monomers in reassembly reactions in part A. **(C) **Native PAGE analysis of adiponectin oligomers after collapse of octadecamers to hexamers at pH 4 and re-neutralization by dialysis against PBS followed by subsequent treatment with either 5 mM βME or PBS as control. **(D) **Non-reducing denaturing SDS-PAGE analysis of redox states of adiponectin oligomers in part C. Samples were removed and treated with NEM at the various time points to quench reactions.

To further assess if disulfide rearrangement leads to oligomerization into HMW adiponectin, we first prepared oxidized hexamers by reducing the pH of the purified octadecamers to 4 in absence of reducing agents. Subsequently, varying concentrations of βME were added to partially reduce adiponectin and allow disulfide rearrangement to occur. After amounts of reduced adiponectin reach equilibrium following addition of βME, any oligomerization that takes place in absence of net oxidation or reduction is most likely the result of disulfide rearrangement. Interestingly, 100 μM βME led to immediate reduction of small amounts of adiponectin without promoting immediate octadecamer formation (Figure 5A and 5B). However, after 60 min of incubation, the octadecamer began to assemble, and continued to increase until 240 min (Figure 5A). During this time period the concentrations of oxidized dimers and reduced monomers remained constant (Figure 5B), indicating lack of net oxidation or reduction. Similar patterns of adiponectin assembly and oxidation were observed with the addition of 500 μM βME (Figure 5A and 5B). At 5 mM βME, HMW species re-oligomerized within 5 min (Figure 5A).

The hexamers in Figure 5A and 5B migrated in a diffuse manner in native gels. In contrast to previous experiments in which the glycine used for octadecamer collapse was either removed by dialysis or diluted in PBS, the combination of the use of Tris-base to neutralize the reactions and the presence of high concentrations of glycine likely caused hexamers to migrate diffusely. To demonstrate that hexamers indeed decreased at the expense of HMW and trimeric adiponectin following reduction by βME, separate experiments were performed in which the reactions were dialyzed against PBS at pH 7 for 3 hrs prior to addition of βME. As shown in Figure 5C and 5D, upon neutralization by dialysis, 5 mM βME led to decreased levels of hexamers along with increased octadecamers and trimers, indicating reduction of hexamers and reassembly of adiponectin octadecamers. In general, increased proportions of reduced adiponectin resulted in faster re-oligomerization of HMW adiponectin and resulted in higher levels of octadecameric and trimeric species at the expense of hexamers, pointing to disulfide rearrangement facilitating adiponectin oligomerization.

## Discussion

Low serum levels of HMW adiponectin are associated with obesity and insulin resistance [12-16]. It has been suggested that this selective decrease is due to a suboptimal folding environment in the ER [26]. We have attempted to analyze the *de novo *formation of HMW adiponectin from the trimer in order to build a detailed model of adiponectin assembly that could explain *in vivo *data at molecular detail and to suggest additional cell-based experiments to validate the model. Recently, we reported that oligomerization to HMW adiponectin occurs through partially reduced intermediates stabilized by disulfide bonds [21]. The results led us to postulate that excessively oxidizing conditions would accelerate disulfide bonding and favor the formation of fully oxidized hexamers that are unable to assemble further into HMW adiponectin. Conversely, an optimal redox condition that balances a moderately reducing redox potential with a sufficiently rapid rate of oxidation would lead to preferential accumulation of HMW adiponectin in a timely manner. To test these hypotheses, we monitored oligomerization of adiponectin under various redox conditions. Surprisingly, we did not find such an optimal redox condition. Redox potentials were either too low to lead to significant oxidative assembly in a physiological relevant time frame or too high to generate high amounts of HMW adiponectin. Large amounts of HMW adiponectin were possible only when reassembly reactions were carried out under oxidizing conditions in which disulfide rearrangement could occur rapidly.

Several lines of evidence indicate that disulfide rearrangement is crucial for assembly of HMW adiponectin. First, increasing total glutathione concentrations under oxidizing conditions led to increased formation of HMW adiponectin (Figure 4). Second, hexamers unable to oligomerize into HMW adiponectin could do so after some of the disulfide bonds in hexamers were reduced, resulting in appearance of trimers and octadecamers (Figure 5). Third, majority of intracellular adiponectin exists as hexamers or trimers [20]. It is possible that disulfide rearrangement represents the rate-limiting step in converting hexamers to HMW species. Lastly, whereas slow rates of disulfide bond formation during gradual removal of reducing agents led to robust reassembly of HMW adiponectin (Figure 1), complete removal of reducing agents followed by rapid oxidation favored hexamers (Figures 2 and Additional file 1: Figure S1). In our previous study, reassembly of large amount of HMW adiponectin was possible because the oxidizing agent used in many of the experiments, hydrogen peroxide, was introduced into reactions slowly by dialysis [21]. Rapid disulfide exchange was possible under these conditions in which oxidation occurred slowly at first but allowed to reach completion following complete removal of reducing agents.

Under highly oxidizing conditions, sizable amounts of trimers were unable to oligomerize further into either hexamers or HMW adiponectin (Figures 3 and Additional file 1: Figure S1). These trimers likely contain intra-trimer disulfide bonds that do not allow formation of higher-order oligomers. Adiponectin contains a collagen domain that forms a triple helix among three monomers, although the architecture of the collagen domains in the higher-order adiponectin oligomers remains unknown. Formation of a disulfide bond within a trimer could lock the conformation of the collagen domain and limits the flexibility of the triple helical coil at the N-terminal end of the molecule. If an intra-trimer disulfide bond forms before an inter-trimer disulfide bond, further oligomerization may be halted. Alternatively, if inter-trimer disulfide bonds form before intra-trimer disulfide bonds, then additional oligomerization could take place. Disulfide rearrangement could facilitate oligomerization by exchanging intra-trimer disulfide bonds with inter-trimer disulfide bonds.

In addition to conformational constraints described above, the inability of adiponectin trimers to assemble into larger oligomers could be caused by inaccessibility of the unpaired cysteine near the N-terminus to solvent. While direct evidence in support of this hypothesis is currently lacking, prior work indicates that isolated trimers do not assemble into larger species spontaneously. We have previously transfected HEK 293T cells with mouse adiponectin cDNA in an expression vector and purified adiponectin oligomers secreted from transfected cells [9]. In addition to hexamers and octadecamers, trimers were also secreted from transfected 293T cells. These trimers were shown to consist of one monomer and one disulfide-bonded dimer [10] and did not convert to another oligomer following purification by ion exchange and gel filtration chromatography [9]. In addition, it was previously shown that when semi-purified human serum adiponectin trimers were injected into rabbit veins, there was no conversion to other oligomers in blood after 30 hrs [27].

Even though redox conditions were relatively reducing in both Figure 5 (after βME addition) and the rightmost panel of Figure 2, the distribution of oligomers differed. Rearrangement of hexamers in Figure 5 led to predominantly octadecamers while reassembly from trimers at -200 and -220 mV did not lead to significant amounts of octadecamers (Figure 2A). This likely reflects kinetic differences in the rate of oligomerization. When two hexamers collide and undergo disulfide rearrangement, the products are likely one nonamer (9 mer) and one trimer. In general, disulfide rearrangements between a growing oligomer with a hexamer will result in an even larger oligomer and a trimer. This is supported by results in Figure 5 in which the assembly of octadecamers from hexamers invariably led to formation of trimers. In contrast, when a growing oligomer collides with a trimer and undergoes rearrangement (as in Figure 2A), the products are a rearranged oligomer of original size and a trimer that used to be part of a larger oligomer. As a result, assembly from hexamers should be significantly faster than from trimers. Given the importance of hexamer as an intermediate in the assembly of octadecamer, redox regulation of hexamer formation is likely a highly important aspect of adiponectin oligomerization.

Results in Figure 1 and in our prior publication [21] led us to propose that self-assembly of adiponectin octadecamers proceeds spontaneously through partially reduced intermediates. This conclusion requires re-examination in light of current results showing trapping of adiponectin oligomerization at trimeric and hexameric states in oxidizing environment (Figures 2 and 3). Adiponectin oligomerization to octadecamers requires disulfide bonds to stabilize intermediate oligomers. Thus the question of whether adiponectin oligomerization is a spontaneous process depends on whether disulfide bond formation in adiponectin is spontaneous. Redox reactions consist of two half-reactions, one oxidizing and the other reducing, thus disulfide formation cannot take place unless oxidizing equivalents are present along with reduced thiols. Disulfide bond formation is spontaneous in the presence of electron acceptors with higher reduction potential. In the adiponectin reassembly and oxidation experiments in which reducing agents were removed gradually by dialysis (Figure 1), the identity of the immediate electron acceptor is not clear although we hypothesize that molecular oxygen was the ultimate electron acceptor. Reduction of molecular oxygen to water has a high standard reduction potential of 0.85 V [28], making oxidation of thiols to disulfides a highly favored and spontaneous process. In subsequent experiments where oxidized glutathione and diamide are electron acceptors (Figures 2 and 3, respectively), we observed that presence of electron acceptors with high reduction potential is not sufficient for oligomerization to octadecamer. Assembly of adiponectin likely follows the well-established free energy funnel paradigm for protein folding [29,30]. As an intermediate oligomer grows by forming disulfide bonds with additional subunits, it may fall into one of the numerous local minima in the energy landscape. It is possible that to get out from a low energy trap, an assembly intermediate needs to be reduced once again. In this case oligomerization of adiponectin octadecamers would appear to be spontaneous under conditions where disulfide rearrangement could take place.

These *in vitro *results suggest the need to protect adiponectin from forming intra-trimer disulfide bonds *in vivo*. ERp44 and DsbA-L are ER chaperones that were previously shown to influence adiponectin oligomer distribution [22,24]. ERp44 forms mixed disulfides with adiponectin [24], which could prevent intra-trimer disulfide formation. The role of glutathionylation in increasing oligomerization of the octadecamer under oxidizing conditions (Figure 4) could be analogous to the role of ERp44 in forming mixed disulfides with adiponectin *in vivo*. DsbA-L contains a SXXS motif in its thioredoxin-like domain instead of CXXC [22]. The hydroxyl groups in DsbA-L may protect thiol groups from adjacent adiponectin monomers from forming a disulfide bond. Decreased production of total and HMW adiponectin associated with insulin resistance may be due in part to decreased cellular levels of ERp44 and DsbA-L. A 50% reduction in serum HMW adiponectin was associated with an approximately 80% decrease in adipose tissue ERp44 in the genetically obese and insulin-resistant *ob*/*ob *mice [24]. Decreased serum and adipose tissue adiponectin were also associated with decreased amounts of adipose tissue DsbA-L protein and mRNA in mice fed a high-fat diet [22]. In a small cohort of humans, total serum adiponectin concentrations were positively correlated with adipose tissue DsbA-L mRNA expression and protein levels of DsbA-L were found to be reduced in obese and overweight individuals [22]. In contrast, treatment with peroxisome proliferator-activated receptor-gamma (PPARγ) agonists, commonly prescribed anti-diabetic medication known to increase insulin sensitivity and serum HMW adiponectin [16], increased ERp44 and DsbA-L protein concentrations in, respectively, mouse adipose tissue [24] and cultured adipocytes [22].

It is unclear at the present if decreased levels of ERp44 and DsbA-L associated with obesity and insulin resistance represent particular manifestations of overall dysfunction in the ER compartment. ER stress characterized by induction of unfolded protein response takes place in adipocytes and hepatocytes of obese animals and contributes to the development of insulin resistance [31]. It is possible that obesity-related decrease in HMW adiponectin production is in part due to ER stress independent of changes in ERp44 or DsbA-L. This could occur as chaperones like BiP are tasked to shield increasing amounts of unfolded proteins and making them unavailable to assist in the proper folding of adiponectin. In addition, it was shown recently that onset of pharmacologically- or nutritionally-induced ER stress in yeast led to a more reducing ER redox state [32]. If induction of unfolded protein response in adipocytes is also accompanied by a more reduced ER redox environment, it could affect the rates of disulfide bond formation and/or rearrangement during assembly of HMW adiponectin oligomers. It is possible that impaired adiponectin oligomerization associated with insulin resistance is linked by an altered ER redox state.

## Conclusions

This report further analyzes the role of disulfide bond formation in adiponectin oligomerization. Our results emphasize the importance of conditions that promote inter-trimer disulfide bonds, including an oxidizing redox environment with sufficient amounts of redox couples for disulfide rearrangement, suggesting that regulation of redox environment in adipocytes is a potential target for future treatments against type 2 diabetes.

## Methods

### Oligomerization state of reassembled adiponectin following dialysis into defined concentrations of DTT

Adiponectin octadecamers with minor amounts of hexamers were purified from bovine serum as previously described [21]. Purified adiponectin at 0.25 mg/mL was treated with 5 mM dithiothreitol (DTT) for 2 hrs at 37°C, followed by addition of glycine (final concentration 50 mM) to lower the pH to around 4 and further incubated for 30 min at 37°C to collapse to trimers. The samples were then placed in MINI-Dialysis units (Pierce, Rockford, IL) and dialyzed into PBS at pH 7.6 containing 5, 1, 0.2, 0.04, or 0.008 mM DTT. Samples were removed for oligomerization and oxidation state analysis using native and denaturing PAGE as described previously [21] after 6 and 18 hrs of dialysis. Dialysis buffer was replenished at 6, 12, and 18 hrs with newly formulated buffer containing fresh DTT. For native PAGE analysis, samples were diluted in concentrated native gel loading buffer and immediately subjected to electrophoresis or treated with 10 mM thiol-inactivating agent *N*-ethylmaleimide (NEM) for 20 min at 37°C and frozen at -20°C until electrophoresis. For non-reducing denaturing PAGE analysis, samples were treated with 10 mM NEM at 37°C for 20 min and frozen at -20°C until electrophoresis. Samples were denatured at 85°C for 20 min in SDS-PAGE gel loading buffer without reducing agents. The assignments of trimers, hexamers, and octadecamers in native gels were independently confirmed using native mass spectrometry [21] and gel filtration chromatography [9]. The oligomerization states of nonamers (9 mers) and dodecamers (12 mers) were extrapolated from linear fitting of graphs in which the migration distances of the known trimers, hexamers, and octadecamers in native gels were plotted against the natural log of monomer number.

### Oligomerization state of adiponectin reassembled in glutathione-based redox buffers

Purified octadecameric adiponectin was first reduced in 10 mM DTT for 1 hr at 37°C. DTT was then removed by 6 rounds of buffer exchange into PBS supplemented with 5 mM reduced glutathione (GSH) using 30 kD molecular weight cut off Amicon Ultra 0.5 mL centrifugal filter units (Millipore, Billerica, MA). To completely collapse 18 mers to trimers, the DTT-free samples were then incubated in 50 mM glycine at pH 4 for at least 15 min at room temperature. The samples were subsequently neutralized back to pH 7 by a 12.8-fold dilution in PBS to restore adiponectin concentration to 0.25 mg/mL. Following neutralization, aliquots were placed in different ratios of GSH and oxidized glutathione (GSSG) corresponding to reduction potentials of -120, -140, -160, -180, -200, and -220 mV. The Nernst equation for GSH/GSSG couple, E = E°' - (RT/nF)([GSH]^2^/[GSSG]), was applied to calculate the ratios of GSH and GSSG needed to maintain reactions at specific reduction potentials. The value of -240 mV was used for the GSH/GSSG couple standard reduction potential [33]. The total cysteine equivalents in each reaction was between 1 to 3 mM. The samples were incubated at 25°C for the time indicated until the ratios of dimers to monomers reached equilibrium. Upon proceeding to completion, the reactions were quenched in 10 mM NEM and stored at -20°C until analyses. The portion of the procedure from DTT removal to reaction quenching by NEM was carried out in an anaerobic chamber (Coy Laboratory Products, Grass Lake, MI) using degassed buffers to minimize oxidation by atmospheric oxygen. Experiments in which the reduction potentials were stretched from -155 mV to +40 mV differed from the rest in two aspects: DTT was removed by dialysis into PBS at pH 7.6 and total concentrations of glutathione were 10 mM.

To assess the effect of glutathione concentration on adiponectin oligomerization, reduced trimers were prepared following removal of DTT using centrifugal filter units as described above and incubated in 2, 5, 10, or 15 mM total glutathione for indicated amounts of time at room temperature with reduction potential maintained at -120 mV. In addition to oligomerization and oxidation state analysis by PAGE and Coomassie staining, aliquots of the reactions were subjected to non-reducing denaturing SDS-PAGE, transferred to nitrocellulose membranes, and probed for glutathionylation of adiponectin using an anti-GSH primary antibody (Arbor Assays, Ann Arbor, MI) followed by a an infrared conjugated secondary antibody (LI-COR Biosciences, Lincoln, NE)

### Oligomerization state of adiponectin reassembled in presence of diamide

Purified adiponectin was collapsed to trimers by sequential incubations at 37°C in 10 mM DTT for 1 hr at pH 7 and 50 mM glycine for 30 min at pH 4. The samples were neutralized by dilution in PBS and diamide was added to experimental groups at a final concentration of 25 mM prior to dialysis against PBS. Equal volumes of 20 mM NEM were added at the end of each time point. Oligomerization and oxidation states were determined as described above.

### Reassembly of HMW adiponectin from hexamers treated with βME

Hexamers were generated by treating purified HMW adiponectin with 50 mM glycine at pH 4 and incubated at 37°C for at least 15 min. Upon neutralization in Tris-base, βME was added to the reactions at final concentrations of 100 μM, 500 μM and 5 mM and incubated for 4 hrs at 25°C. At each time point, aliquots were removed for native and non-reducing denaturing PAGE. Samples for non-reducing SDS-PAGE were first treated with 20 mM NEM prior to heat denaturation. In separate experiments, βME-mediated reassembly reactions were performed after the samples were neutralized by dialysis against PBS at pH 7 for 3 hrs instead of addition of Tris-base.

### Native and denaturing PAGE analysis of adiponectin oligomers

Adiponectin oligomers were fractionated in 7% polyacrylamide gels under native conditions as previously described [21]. The oxidation states of the cysteine residue near the N-terminus of adiponectin were determined by non-reducing denaturing PAGE [21]. All experiments were performed minimally three times, and figures presented are representative of the results.

## List of abbreviations

βME - β-mercaptoethanol; DTT - dithiothreitol; ER - endoplasmic reticulum; GSH - reduced glutathione; GSSG - oxidized glutathione; HMW - higher molecular weight; kD - kilodalton; mV - millivolts; NEM - *N-*ethylmaleimide; DsbA-L - Disulfide-bond A oxidoreductase-like protein; ERp44 - ER resident protein 44 kD; TZD - thiazolidinediones; PAGE - polyacrylamide gel electrophoresis; PPAR-γ - peroxisome proliferator-activated receptor-gamma; Tris - tris(hydroxymethyl)aminomethane

## Authors' contributions

DBB designed and carried out the experiments described in Figures 2, 4, 5, and Additional file 1: figure S1 and drafted parts of the manuscript. RMG designed and carried out the experiments described in Figure 3. PRM designed and carried out the experiments described in Figure 1. MN assisted in the design and execution of most re-oligomerization experiments. TST participated in the design and coordination of the overall study and drafted parts of the manuscript. All authors read and approved the final manuscript.

## Supplementary Material

Additional file 1 **Supplemental Figure S1. Redox titration of adiponectin oligomerization in glutathione-based redox buffers with extended range of reduction potential**. The figure and associated legend are combined in a single PDF file. The complete file name is Figure S1.pdf click here for file
