# Supplementary material for: Role of redox environment on the oligomerization of higher molecular weight adiponectin
Source: BMC Biochem. 2011 May 23;12:24. doi: 10.1186/1471-2091-12-24 (PMC3117782; doi:10.1186/1471-2091-12-24)
Supplement: Additional file 1 — Supplemental Figure S1. Redox titration of adiponectin oligomerization in glutathione-based redox buffers with extended range of reduction potential. The figure and associated legend are combined in a single PDF file. The complete file name is Figure S1.pdf [file 1471-2091-12-24-S1.PDF]

Supplementary Information

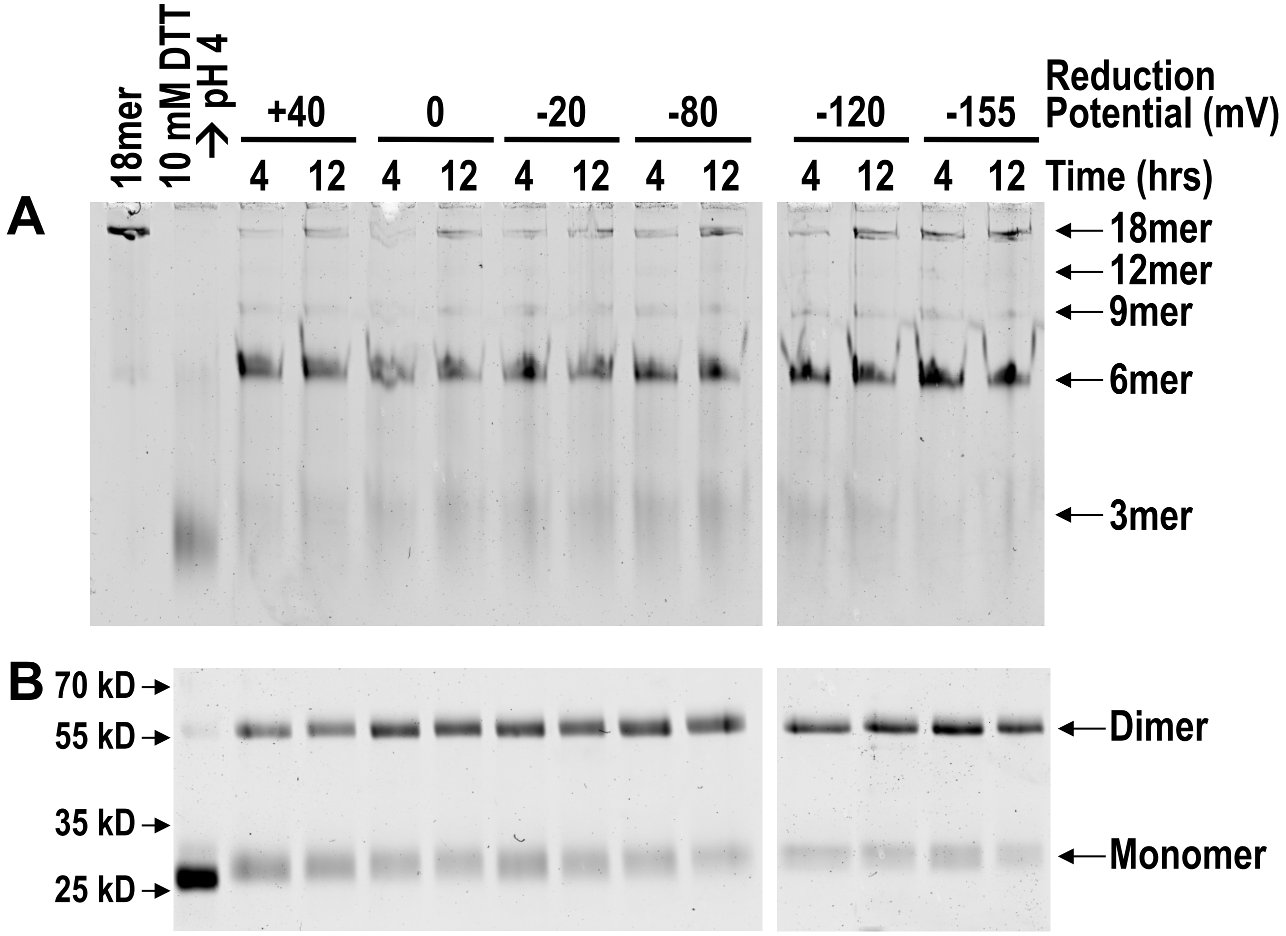

Supplemental Figure S1

Supplemental Figure Legend

Figure S1. Redox titration of adiponectin oligomerization in glutathione-based redox buffers with extended range of reduction potential. **(A)** Native PAGE of adiponectin oligomers after collapse to trimers and subsequent incubation at the various glutathione-based reduction potentials for the indicated periods of time and **(B)** Non-reducing denaturing SDS-PAGE of disulfide-bonded dimers and reduced monomers in different reduction potentials. Purified octadecameric adiponectin was collapsed to trimers using DTT followed by lowering of pH to 4. Subsequently, the samples were neutralized and DTT removed by dialysis. The samples were then incubated in various glutathione-based redox buffers with reduction potentials ranging from -155 mV to +40 mV for 4 and 12 hrs at room temperature. The total glutathione concentration for these experiments was 10 mM. To decrease oxidation by atmospheric oxygen, the experiments were performed in an anaerobic glove box. At each time points, aliquots were removed and quenched using NEM.
